# Mitochondrial Quality Control via Mitochondrial Unfolded Protein Response (mtUPR) in Ageing and Neurodegenerative Diseases

**DOI:** 10.3390/biom13121789

**Published:** 2023-12-13

**Authors:** Paula Cilleros-Holgado, David Gómez-Fernández, Rocío Piñero-Pérez, Jose Manuel Romero-Domínguez, Diana Reche-López, Alejandra López-Cabrera, Mónica Álvarez-Córdoba, Manuel Munuera-Cabeza, Marta Talaverón-Rey, Alejandra Suárez-Carrillo, Ana Romero-González, Jose Antonio Sánchez-Alcázar

**Affiliations:** Centro Andaluz de Biología del Desarrollo (CABD-CSIC-Universidad Pablo de Olavide), 41013 Sevilla, Spain; pcilhol@upo.es (P.C.-H.); dgomfer1@acu.upo.es (D.G.-F.); rpieper@alu.upo.es (R.P.-P.); jmromdon@upo.es (J.M.R.-D.); dreclop@alu.upo.es (D.R.-L.); alopcab2@alu.upo.es (A.L.-C.); malvcor@upo.es (M.Á.-C.); mmuncab@upo.es (M.M.-C.); mtalrey@upo.es (M.T.-R.); asuacar@alu.upo.es (A.S.-C.); aromgon1@upo.es (A.R.-G.)

**Keywords:** mitochondria, mitochondrial quality control mechanisms, mitochondrial biogenesis, mitochondrial dynamics, mitophagy, mitochondrial unfolded protein response (mtUPR), mitochondrial diseases, neurodegenerative diseases, therapeutic target, ageing

## Abstract

Mitochondria play a key role in cellular functions, including energy production and oxidative stress regulation. For this reason, maintaining mitochondrial homeostasis and proteostasis (homeostasis of the proteome) is essential for cellular health. Therefore, there are different mitochondrial quality control mechanisms, such as mitochondrial biogenesis, mitochondrial dynamics, mitochondrial-derived vesicles (MDVs), mitophagy, or mitochondrial unfolded protein response (mtUPR). The last item is a stress response that occurs when stress is present within mitochondria and, especially, when the accumulation of unfolded and misfolded proteins in the mitochondrial matrix surpasses the folding capacity of the mitochondrion. In response to this, molecular chaperones and proteases as well as the mitochondrial antioxidant system are activated to restore mitochondrial proteostasis and cellular function. In disease contexts, mtUPR modulation holds therapeutic potential by mitigating mitochondrial dysfunction. In particular, in the case of neurodegenerative diseases, such as primary mitochondrial diseases, Alzheimer’s disease (AD), Parkinson’s disease (PD), Huntington’s disease (HD), Amyotrophic Lateral Sclerosis (ALS), or Friedreich’s Ataxia (FA), there is a wealth of evidence demonstrating that the modulation of mtUPR helps to reduce neurodegeneration and its associated symptoms in various cellular and animal models. These findings underscore mtUPR’s role as a promising therapeutic target in combating these devastating disorders.

## 1. Introduction

Mitochondria are double-membrane organelles that originated from symbiosis between an α-proteobacteria-like progenitor and a pre-eukaryotic cell [1]. The most common function of mitochondria is their role in energy metabolism. Reducing equivalents are formed during the tricarboxylic acid (TCA) cycle and are subsequently used by the mitochondrial electron transport chain (mtETC) to generate a proton gradient, which is finally used for ATP formation in the process known as oxidative phosphorylation (OXPHOS) [2,3]. Beyond energy production, mitochondria are also involved in other functions such as fatty acid synthesis [4], iron sulfur cluster [5] and heme biogenesis [6], calcium homeostasis [7], apoptosis regulation [8], amino acid and lipid biosynthesis [9,10], etc. All these functions are carried out by mitochondrial proteins. The mitochondrial proteome is composed of about 1500 proteins, although only 13 of them are encoded by mitochondrial DNA (mtDNA). The rest are encoded by nuclear DNA (nDNA), synthesized on cytosolic ribosomes as precursors, and must be transported to the mitochondria [11]. The fact that mitochondrial function depends on both genomes necessitates precise coordination and communication between them.

Mutations in mitochondrial proteins encoded by mtDNA or nDNA, the overproduction of reactive oxygen species (ROS), the deregulation of import processes, or an uncoupled mtETC lead to a disruption in mitochondrial function, which is responsible for primary mitochondrial diseases [12] and, secondarily, for other illnesses and physiological processes related to mitochondrial dysfunction, such as neurodegenerative diseases [13], ageing [14], metabolic diseases [15], cardiac disorders [16], and cancer [17]. 

To ensure the correct functioning of mitochondria and cells in general, cells employ different protein quality control mechanisms to prevent mitochondrial perturbations. In this review, we will summarize these mechanisms, beginning with mitochondrial biogenesis and the cytosolic quality control of mitochondrial protein import. We will then proceed to mitochondrial dynamics, encompassing fusion and fission processes, followed by mitophagy, with special attention paid to the role of the mitochondrial unfolded protein response (mtUPR) and its therapeutic potential.

## 2. Mitochondrial Quality Control

Since mitochondria are essential for eukaryotic organisms, these organelles possess several quality control mechanisms for counteracting dysregulated mitochondrial activity. Among them, the cytosolic protein quality control system prevents abnormal polypeptides from entering the mitochondria during mitochondrial biogenesis [18]. Mitophagy removes parts or entire mitochondria in response to mitochondrial damage [19]. Mitochondrial-derived vesicles (MDVs) are generated when mitochondrial membranes become oxidized due to mitochondrial stress, causing them to be loaded into vesicles, which are then transferred to lysosomes or peroxisomes for degradation [20]. The mtUPR induces the activation of mitochondrial chaperones and proteases, as well as antioxidant enzymes, to counteract mitochondrial stress [21]. Eventually, mitochondrial dynamics, including fusion and fission processes, regulate mitochondrial homeostasis [22].

### 2.1. Mitochondrial Biogenesis

Mitochondrial biogenesis requires special coordination between nuclear and mitochondrial genomes. It is defined as a self-renewal pathway by which new mitochondria are formed from pre-existing ones [23]. Mitochondrial biogenesis involves two different subprocesses: (1) mtDNA transcription and translation and (2) the transcription, translation, import, and assembly of nDNA-encoded mitochondrial proteins [24].

mtDNA transcription is activated by the family of peroxisome proliferator-activated receptors, among which peroxisome proliferator-activated receptor γ co-activator 1α (PGC-1α) is considered the master regulator [25]. PGC-1α can be activated via phosphorylation through AMP-activated kinase (AMPK) or deacetylation via SIRT1 [26]. PGC-1α can also be activated by cAMP response element-binding protein (CREB), which, in turn, is activated through phosphorylation via protein kinase A (PKA) [27], calcium-calcium/calmodulin-dependent protein kinase (CaMK) [28], or protein kinase B, also known as Akt [29]. The activation of PGC-1α is followed by the stimulation of nuclear respiratory factors 1 and 2 (NRF1 and NRF2), which promote the transcription of multiple mitochondrial genes, including mitochondrial transcription factor A (TFAM), the first well-characterized transcription factor from mammalian mitochondria. This leads to mtDNA replication and transcription [30]. Finally, the translation of the mtDNA-encoded genes into proteins takes place.

On the other hand, nDNA-encoded mitochondrial proteins are synthesized on cytosolic ribosomes as precursors in response to the activation of nuclear transcription factors such as NRFs. These preproteins traverse the outer mitochondrial membrane (OMM) through the translocase of the outer membrane (TOM) complex system, which recognizes internal and cleavage mitochondrial targeting signal (MTS) present in the N-terminal [31]. Then, the precursors cross the inner mitochondrial membrane (IMM) because of the positive charge present in their MTS, which initiates the electrophoretic translocation process. This process involves their entry into the mitochondria through the translocase of the inner membrane (TIM) complex, relying on the electric potential across the IMM [32]. Subsequently, mitochondrial precursors are transported to the mitochondrial matrix with the assistance of mitochondrial chaperones, which facilitate import and folding [33]. Finally, proteins undergo proteolytic removal of their MTS sequence, an important step in their maturation, and attain their final conformation through the action of mitochondrial chaperones [34]. There are other specific import pathways depending on the location of the mitochondrial proteins. For more information, various articles are available for reference [35,36,37,38].

**Figure 1:**
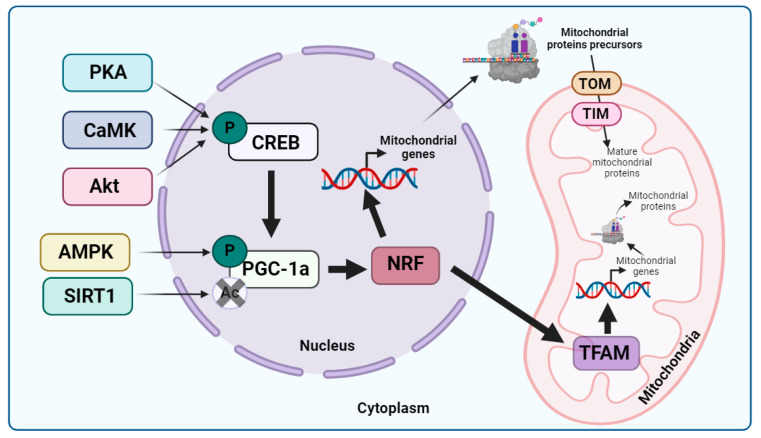
Mitochondrial biogenesis. Considering that PGC-1α is the main regulator of mitochondrial biogenesis, it can be activated through phosphorylation via CREB, which, in turn, is phosphorylated by PKA, CaMK, or Akt, or via AMPK. PGC-1α can also be activated via deacetylation through SIRT1. Once this transcription factor is activated, it stimulates NRF transcription factors that activate the transcription of nDNA-encoded mitochondrial genes and TFAM. Subsequently, TFAM promotes mtDNA replication and transcription, while mitochondrial proteins encoded by nDNA are synthesized on cytosolic ribosomes as precursors to allow them to cross the OMM and IMM through the TOM and TIM systems, respectively. Once in the mitochondrial matrix, the mitochondrial precursors undergo proteolytic processing necessary for their maturation, reaching their final conformation due to the action of mitochondrial chaperones. Arrow: activation or induction of the subsequent process.

Therefore, it is necessary to synchronize the transcription and translation of two different and physically separated genomes for mitochondrial biogenesis, something that occurs due to the action of PGC-1α and NRF transcription factors (see Figure 1).

Mitochondrial biogenesis can be activated by various stimuli, such as exercise, diet, hormones, and stressors. In the last case, mitochondrial biogenesis might be upregulated to enhance mitochondrial function or to compensate for the increased rates of mitophagy [39].

#### Cytosolic Quality Control of Mitochondrial Protein Import

During mitochondrial biogenesis, mitochondrial proteins precursors synthesized on cytosolic ribosomes can exceed the capacity of the mitochondrial translocation machinery, leading to an accumulation of non-imported mitochondrial proteins precursors in the cytoplasm and, consequently, proteotoxic stress [40]. This accumulation, named mitochondrial precursor overaccumulation stress (mPOS), can be alleviated through several mechanisms [41]. The immediate response triggered is known as the unfolded protein response activated by the mistargeting of proteins (UPRam), which involves the assembly of the proteasome through the factors Irc24 and Poc4 [42]. This leads to an increase in proteasomal activity that degrades mitochondrial precursors in the cytosol, contributing to a response known as the mitoprotein-induced stress response. The latter is dependent on heat shock transcription factor 1 (HSF1), which, under stress conditions, is released from chaperone complexes [18].

On the other hand, the mitoprotein-induced stress response leads to a decrease in translation activity through the phosphorylation of the eukaryotic translation initiation factor 2α (eif2α) [43]. Thus, the increased degradation activity and decreased translation promote the clearance of mitochondrial protein precursors, reducing proteotoxic stress. Moreover, the cytosolic quality control of mitochondrial protein import is strongly associated with the mtUPR, since ATFS-1 in *C. elegans* and ATF5 in mammalian cells are both MTSs and nuclear localization signals (NLSs). When mitochondrial import fails, these factors are transported to the nucleus, where they promote the transcription of molecular chaperones and proteases, leading to mtUPR activation [42].

### 2.2. Mitochondrial Dynamics

Mitochondrial dynamics are defined as a balance between fission and fusion processes [22] that change mitochondrial shape and size under pathological and physiological conditions. These processes are responsible for mtDNA stability, mitochondrial morphology and homeostasis maintenance, mitochondrial respiratory capacity, and response to cellular stress [44]. Therefore, mitochondrial fusion and fission are part of the quality control system, and the disruption of these processes is the cause of several human diseases.

Mitochondrial fusion is defined as the merging of two mitochondria into one [45]. For this process to transpire, three enzymes of the dynamin-like family of GTPases are required: Mitofusins 1 and 2 (Mfn1, Mfn2), located in the OMM, and Optic Atrophy 1 (Opa1), anchored in the IMM [46]. Mitochondrial fusion begins with the interaction of the mitofusins present in the two fusing mitochondria, which form homo- or hetero-oligomers [47]. After that, Opa1 mediates the fusion of the IMMs of both mitochondria. However, unlike in the case of mitofusins, Opa1 must only be present in one of the two fusing mitochondria [48]. Finally, the contents of both mitochondria mix, giving rise to a new larger mitochondrion with the mitochondrial matrix components of the two parent mitochondria diffused throughout it, including mtDNA [49]. Therefore, mitochondrial fusion can aid in the rescue of damaged mitochondria by diluting aberrant proteins or allowing wild-type mtDNA to compensate for flaws [50].

Mitochondrial fusion can be triggered in response to several factors, such as exposure to ultraviolet C (UV-C) irradiation, hydrogen peroxide, cycloheximide, actinomycin D, or azinomycin or starvation, among others. It leads to hyperfusion, a process centered on maintaining ATP levels within mitochondria and responding to stress in a pro-survival process known as stress-induced mitochondrial hyperfusion (SIMH) [51]. In this process, stromatin-like protein 2 (SLP-2) is required, as when SLP-2-depleted cells are exposed to mitochondrial stress, mitochondria do not become hyperfused but fragmented. This is due to the requirement of SLP-2 to prevent the proteolytic cleavage of Opa1, indicating that long isoforms of Opa1 are necessary for the SIMH process [52]. SIMH can also be stimulated by the unfolded protein response in the endoplasmic reticulum (UPR^ER^) in a manner dependent on the phosphorylation of eif2α, one of the key factors in the mtUPR, as we will discuss throughout this review [53].

Additionally, when exposed to a virus, mitochondrial fusion may be activated, and it supports the innate immune response [54].

On the other hand, mitochondrial fission, the opposing process of mitochondrial fusion, is defined as the separation of one mitochondrion into two or more daughter mitochondria [45]. Several proteins are necessary in this process, with dynamin-related protein 1 (Drp1) playing a central role. Drp1 is usually located in the cytosol, from which it translocates to the OMM. Then, Drp1 is anchored to the mitochondrial surface with the help of mitochondrial fission 1 protein (FIS1), mitochondrial dynamics proteins 49 (MID49) and 51 (MID51), or mitochondrial fission factor (MFF) [55]. Once Drp1 is on the OMM, it forms large homomultimeric complexes to facilitate constriction and scission, processes that are dependent on GTP hydrolysis [56]. To ensure the correct segregation of mtDNA, it must be replicated synchronously with fission.

Furthermore, two types of mitochondrial fission have recently been described, namely, ‘peripheral fission’ (less than 25% from an extreme) and ‘midzone fission’ (inside the middle 50%), depending on the Drp1 adaptors involved. In the case of peripheral divisions, FIS1 is the protein involved, not serving as a Drp1 adaptor to the OMM but acting as a recruiter of lysosomes for subsequent mitochondrial degradation through mitophagy. Nevertheless, in the case of midzone fissions, the Drp1 adaptor implicated is MFF. However, the protein involved is not the sole distinction between both types of mitochondrial fission. It has been reported that daughter mitochondria from peripheral divisions are targeted for degradation through mitophagy, while those originating from midzone divisions contribute to mitochondrial biogenesis during cell proliferation [57].

Mitochondrial fission can be activated after shorts periods of exercise due to increased ROS levels. However, after the recovery period, mitochondria become increasingly networked, implying that the activation of mitochondrial fission acts as a process of acclimation to an exercise state [58].

In general terms, mitochondrial fusion is activated in response to low-level or chronic exposure to stressors, while mitochondrial fission is stimulated when there is acute high-level exposure [59]. Nevertheless, if mitochondrial damage cannot be repaired via mitochondrial dynamics, the mitochondria are eliminated via mitophagy, the selective autophagy of mitochondria.

### 2.3. Mitophagy

**Figure 2:**
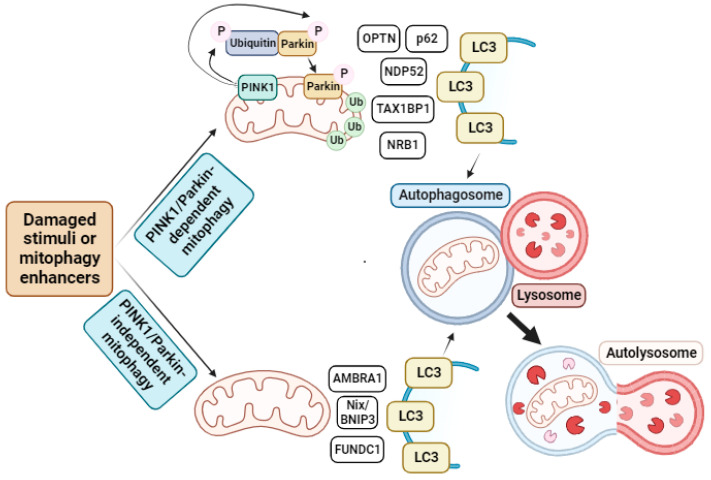
Mitophagy pathway. In response to damaged stimuli or through mitophagy enhancer supplementation, cells activate mitophagy. This process can be divided into two different subprocesses depending on the participation of PINK1 and Parkin proteins. In the PINK1/Parkin-dependent cascade, PINK1 at the OMM is activated and phosphorylates Parkin. However, this phosphorylation is not sufficient to recruit Parkin to the OMM. On the contrary, PINK1 phosphorylates ubiquitin, which binds to Parkin, facilitating its recruitment to the mitochondria. Then, Parkin ubiquitinates several proteins on the mitochondrial surface, which are recognized by different receptors such as OPTN, p62, NDP52, TAX1BP1, or NRB1 that, in turn, interact with LC3 present in the autophagosome membrane and stimulate the formation of autophagosome. These autophagosome fuse with lysosomes, leading to the formation of autolysosomes, where mitochondrial degradation occurs. On the other hand, in PINK1/Parkin-independent mitophagy, damaged mitochondria are recognized by other receptors such as AMBRA1, Nix/BNIP3, or FUNDC1 that, as in the previous case, interact with LC3 to form the autophagosome, which again fuses with a lysosome to form the autolysosome, where complete degradation of the mitochondrion take place. Arrow: activation or induction of the subsequent process.

Mitophagy is defined as the specific autophagic elimination of mitochondria [60]. This process was first identified in mammalian cells through electron microscopy studies, which revealed enhanced mitochondrial sequestration in lysosomes after glucagon-stimulated hepatocyte catabolism [61]. When mitochondria become damaged, the IMM experiences prolonged depolarization, leading to the stabilization of the PTEN-induced kinase 1 (PINK1) protein at the OMM. PINK1 is a protein with an MTS, a transmembrane domain, and a kinase domain. When PINK1 is transported to healthy mitochondria, the MTS is processed by mitochondrial protease-like (MPP) protein in the mitochondrial matrix, and the transmembrane segment is processed by the presenilin-associated rhomboid-like (PARL) protein in the IMM. Consequently, PINK1 is degraded [62]. Nevertheless, when PINK1 is transported to damaged mitochondria, its processing does not occur, leading to the accumulation of its long isoform on the OMM, forming a supercomplex of 850 kDa that executes autophosphorylation [63]. Once PINK1 is activated, it phosphorylates the E3 ubiquitin ligase Parkin. However, this phosphorylation alone is not sufficient to recruit Parkin to the mitochondria and activate it. On the contrary, it has been described that PINK1 phosphorylates ubiquitin, which binds to Parkin, facilitating its recruitment to the OMM and activating its ubiquitin ligase activity [64]. Once Parkin is activated, it ubiquitinates multiple proteins on the mitochondrial surface, which are subsequently detected by ubiquitin-binding proteins such as optineurin (OPTN), p62, NDP52, Tax1-binding protein 1 (TAX1BP1), and NRB1. These proteins interact with the microtubule-associated protein 1A/1B light chain 3 (LC3) protein to form autophagosomes [65], which transport the enclosed materials to the lysosome for extensive degradation via lysosomal hydrolases, a fusion stage mediated by Pleckstrin homology domain-containing family M member 1 (PLEKHM1) and HOPS LC3-binding proteins, and the lysosome membrane-associated protein Rab7 [66]. In addition to this classical mitophagy pathway (PINK1/Parkin-related mitophagy), several other mitophagy receptors have been identified in PINK1/Parkin-independent mitophagy, including Fun14 domain-containing protein 1 (FUNDC1), autophagy and beclin 1 regulator 1 (AMBRA1), and Nix/BCL2/adenovirus E1B 19 kDa protein-interacting protein 3 (BNIP3). AMBRA1 has been proposed to aid in the removal of damaged mitochondria by guiding them to autophagosomes through its interaction with LC3 [67]. Nix/BNIP3, on the other hand, is critical for the elimination of mitochondria during erythroid maturation [68]. Finally, FUNDC1, physically interacts with LC3, increasing mitophagic flux [69] (see Figure 2).

Several mitophagy stimulators have been described, such as hypoxia [70], starvation, caloric restriction, and exercise [71], as well as compounds like urolithin A [72], actinonin [73], NAD^+^-boosters [74], trehalose [75], resveratrol [76], bexarotene [77], β-asarone [78], melatonin [79], spermidine [73], UMI-77 [80], kaempferol and rhapontigenin [81], and metformin [82].

**Figure 3:**
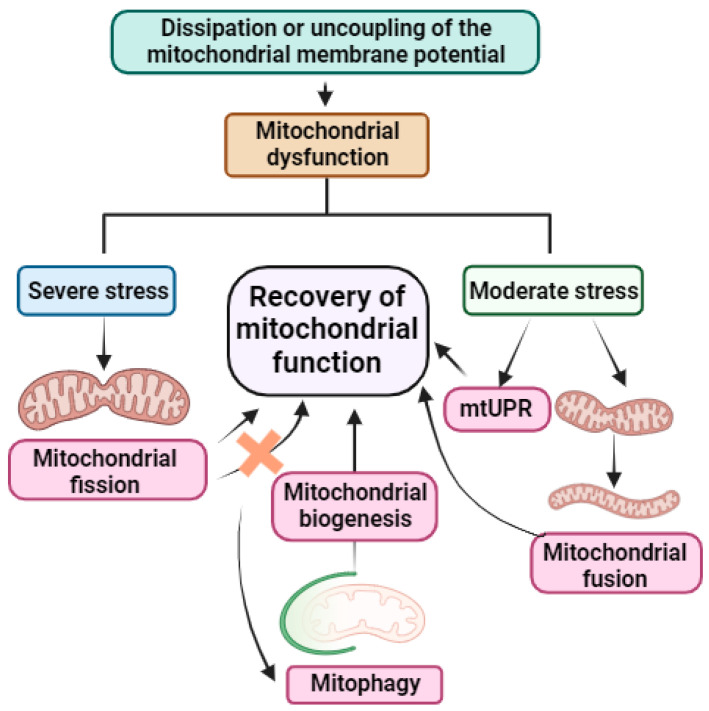
Summary of mitochondrial stress response mechanisms. When mitochondrial dysfunction occurs because of dissipation or uncoupling of mitochondrial membrane potential, mitochondrial protein import fails. This fact is used by cells as a sensor to activate their mitochondrial quality control mechanisms. If the stress is severe, the cells first activate mitochondrial fission. If this does not resolve the stress, cells activate mitophagy, promoting the elimination of damaged mitochondria to preserve those free of damage and inducing mitochondrial biogenesis for the recovery of mitochondrial function. However, if the stress is moderate, cells activate mitochondrial fusion or mtUPR, leading to the recovery of mitochondrial function. Quality control mechanisms of mitochondria are highlighted in pink. Arrow: activation or induction of the subsequent process.

In general, when there is a dissipation or uncoupling of mitochondrial membrane potential, mitochondrial protein import fails, a phenomenon used by cells as a sensor of mitochondrial dysfunction in order to activate their compensatory mechanisms [83]. If the stress is severe, cells first activate mitochondrial fission, and if this is not able to negate the stress, they activate mitophagy [19]. The activation of mitophagy promotes the maintenance of mitochondrial function as it serves as a sensor allowing the cells to induce mitochondrial biogenesis with the aim of maintaining mitochondrial content [84]. However, in situations of moderate stress, cells activate mitochondrial fusion and another mitochondrial quality control process, the mtUPR, leading to the promotion of genes that stimulate mitochondrial function recovery as well as innate immunity and metabolic adaptation, among others [21] (see Figure 3).

### 2.4. Mitochondrial Unfolded Protein Response (mtUPR)

The mtUPR is a vital pathway that maintains mitochondrial homeostasis and proteostasis via stimulating the transcription of nuclear-encoded genes that protect and support mitochondria. This pathway was initially described in rat hepatoma cells by Martinus et al. [85], who discovered that inducing mitochondrial stress through the depletion of mtDNA led to the transcription of mitochondrial molecular chaperones but not those found in other cellular compartments. 

Subsequently, Zhao et al. [86] proposed that the activation of this stress response in mammalian cells, induced by factors such as an ornithine transcarbamylase mutant, not only activates chaperones but also mitochondrial proteases such as ClpP or Lon protease-like 1 (LonP1). Additionally, these researchers identified C/EBP Homologous Protein (CHOP) as a critical transcription factor for mtUPR.

**Figure 4:**
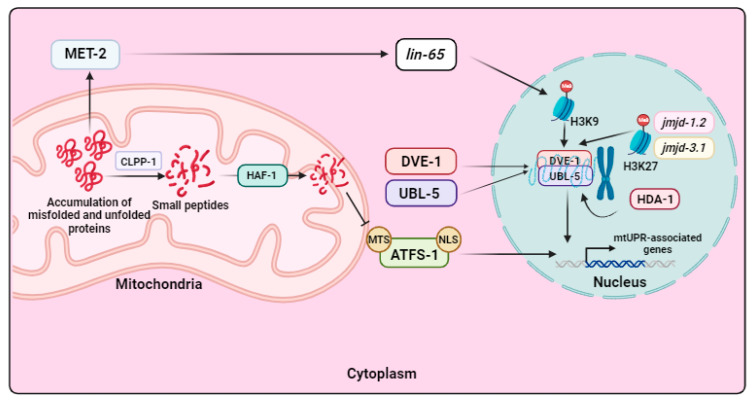
mtUPR signaling in *C. elegans*. When an accumulation of misfolded and unfolded proteins occurs in mitochondria, the CLPP-1 protease is activated and hydrolyzes these proteins into smaller peptides, which are then transported to the IMS by HAF-1. This decreases the transport of the transcription factor ATFS-1 into the mitochondria, causing it to accumulate in the cytoplasm and translocate to the nucleus. In this organelle, ATFS-1 activates the transcription of mtUPR-related genes. Moreover, mtUPR involves two transcription factors, DVE-1 and UBL-5, that, in response to mitochondrial stress, are transported from the cytoplasm to the nucleus, where they form a complex that promotes the transcription of mtUPR-associated genes. In addition, mtUPR is dependent on several mechanisms of chromatin remodeling. Firstly, this stress response pathway is subject to methylation regulation by the histone methylase MET-2, which is activated in response to mitochondrial damage. This activation allows its cofactor LIN-65 to enter the nucleus, where it carries out H3K9 methylation. While most of the chromatin condenses as a result, some regions remain open, and it is in these regions that the complex formed by DVE-1 and UBL-5 binds to promote transcription of mtUPR-related genes. Furthermore, the histone deacetylase HDA-1 also contributes to chromatin decondensation, as do the histone demethylases JMJD-1.2 and JMJD-3.1, allowing for the binding of the DVE-1/UBL-5 complex and triggering the transcription of molecular chaperones and proteases to restore mitochondrial function. Arrow: activation or induction of the subsequent process.

However, the mtUPR has been predominantly studied in *C. elegans.* In this model organism, the process is initiated when there is an accumulation of misfolded or unfolded proteins within the mitochondrial matrix that exceeds the chaperones’ capacity to handle them as well as through other mitochondrial stress-producing factors such as toxins produced by pathogens or mtETC malfunctions, among others [87]. When aberrant proteins accumulate, a complex known as caseinolytic protease P (CLPP-1) hydrolyzes them into short peptides consisting of 8–20 amino acids [88]. Subsequently, the inner-membrane-spanning ATP-binding cassette transporter HAF-1 transports these small peptides from the mitochondrial matrix to the intermembrane space (IMS), reducing the import of the activating transcription factor associated with stress-1 (ATFS-1) into the mitochondria [89]. The activation of response genes and the transfer of mtUPR signals from the mitochondria to the nucleus are both orchestrated by ATFS-1, which contains an NLS and an MTS. Under physiological conditions, ATFS-1 enters the mitochondria using its MTS, where it is degraded by the LonP1 protease. However, during stressful situations, the efficiency of mitochondrial import is compromised, resulting in reduced degradation of ATFS-1 within the mitochondria and its accumulation in the cytosol. This accumulation leads to its transport into the nucleus, where it activates the transcription of mitochondrial molecular chaperones and proteases [90] (see Figure 4).

Additionally, under mitochondrial stress conditions, the homeodomain-containing transcription factor “defective proventriculus” (DVE-1) and ubiquitin-like protein 5 (UBL-5) are translocated into the nucleus, where they form a complex that activates the transcription of mitochondrial chaperones [91]. Finally, chaperones and proteases are transported to the damaged mitochondria to fold or degrade, respectively, unfolded, misfolded, and aberrant proteins [21] (see Figure 4).

Furthermore, it has been described that chromatin remodeling is necessary for the activation of the mtUPR in *C. elegans*. Two histone lysine demethylases are involved in this stress response, namely, JMJD-1.2 and JMJD-3.1. These enzymes lead to the demethylation of H3K27, consequently promoting the transcription of mtUPR-related genes [92]. In addition, the mtUPR is also subject to acetylation regulation. The histone deacetylase HDA-1 collaborates with DVE-1 to activate the transcription of genes linked to mtUPR [93]. Moreover, chromatin remodeling depends on the activity of the histone methyltransferase MET-2 and its nuclear cofactor LIN-65. Generally, methylation globally silences chromatin but leaves some regions accessible, allowing transcription factors to attach themselves. This occurs under mitochondrial stress when MET-2 is activated, promoting LIN-65’s entrance into the nucleus and, as a result, H3K9 methylation. The portions of the chromatin that have not been repressed remain open, and it is in these portions where the UBL-5/DVE-1 complex interacts to enhance the transcription of genes related to the mtUPR [94] (see Figure 4).

It has been described that in *C. elegans*, the mtUPR not only upregulates mitochondrial chaperones and proteases but also promotes the transcription of genes involved in OXPHOS and glycolysis, establishing coordinated regulation of mitochondrial proteostasis and metabolic state [90]. In fact, while mitochondrial damage is being inflicted, ATFS-1 reduces the transcription of OXPHOS and TCA cycle genes while simultaneously promoting the assembly of OXPHOS complexes, leading to an improvement in mitochondrial proteome health [90].

Moreover, Durieux et al. [95] and Berendzen et al. [96] demonstrated that the mtUPR can be activated cell-non-autonomously via mitokines. The presence of mitochondrial stressors within neurons in worms leads to the communication of stress from neuronal mitochondria to peripheral tissues such as the intestine. This process requires serotonin as a mitokine and a functional mtUPR. Consequently, this cell-non-autonomous mode of mtUPR can provide an organism with superior protection against local mitochondrial stressors. 

In mammals, mtUPR regulation is similar to that in nematodes but is far more complex and still not fully understood. Several stressors have been described that can activate the mtUPR in mammalian cells beyond the accumulation of misfolded and unfolded proteins, such as disruptions in OXPHOS, the overproduction of ROS, several compounds, and mitochondrial translation inhibition, among others [97]. In these organisms, a transcription factor ortholog of ATFS-1, known as Activating Transcription Factor 5 (ATF5), has been identified as the main regulator of the mtUPR [98].

Additionally, other transcription factors, including Activating Transcription Factor 4 (ATF4) and CHOP, also play essential roles in regulating the mtUPR in mammals, as they promote the transcriptional expression of ATF5 [99,100,101]. Nevertheless, the communication among these factors is not yet understood. In these organisms, the mtUPR is initiated with the phosphorylation of eif2α, which leads to increased transcription of CHOP, ATF4, and ATF5. These transcription factors are then translocated to the nucleus, where they activate the transcription of mitochondrial chaperones such as Hsp70, Hsp60, and Hsp10, as well as mitochondrial proteases such as ClpP and LonP1 [21,102]. The dependence of the mtUPR on the transcription factors CHOP, ATF4, and ATF5 integrates it with the Integrated Stress Response (ISR), as the primary transcriptional regulator of the latter is ATF4. Moreover, these transcription factors are also shared with UPR^ER^. Both pathways, UPR^ER^ and mtUPR, are responses to stress caused by the accumulation of unfolded or misfolded proteins [103]. However, these processes occur in different cellular compartments, with UPR^ER^ taking place in the endoplasmic reticulum (ER) and mtUPR specifically transpiring in the mitochondria [104]. In fact, one is independent of the other, as the treatment of cells with mitochondrial-specific stressors induced mitochondrial chaperones and proteases without affecting chaperones in the ER. In addition, it has been reported that during mitochondrial stress, ATF5 acts upstream of ATF4, the reverse of what occurs during ER stress [105].

Furthermore, it has recently been described that the proteolytic release of the factor DELE1 by the mitochondrial protease OMA1 carries the signaling of stress from the mitochondria to the cytosol [106], thereby adding a new factor to mtUPR signaling.

Other factors are also implicated in mtUPR regulation in mammalian cells. In situations of mitochondrial stress, the mitochondrial single-stranded DNA binding protein 1 (SSBP1), which is typically found in the mitochondrial matrix, is transported to the cytoplasm via the voltage/dependent anion channel 1 (VDAC1) protein. There, it forms a complex with HSF1, ultimately promoting the transcription of molecular chaperones [107].

It has also been observed that in conditions of mtUPR activation, the amount of MRPP3 protein is reduced, as it is degraded by LonP1. MRPP3 is part of the RNA-free mitochondrial RNase P complex, which also includes MRPP1 and MRPP2, serving as the catalytic subunit of the complex. The reduction in MRPP3 coincides with a decrease in pre-RNA processing following mtUPR induction, leading to a reduction in mtDNA translation, thereby protecting mitochondria from deleterious proteins [108].

Later, in 2018, Münch reviewed other axes of mtUPR [109]. The first is the transcriptional canonical mtUPR axis, which includes the signaling cascade described above, involving the transcription factors CHOP, ATF4, and ATF5, and increased expression of mitochondrial chaperones and proteases [110]. The second is the translational mtUPR axis mentioned earlier, where, in mtUPR induction situations, MRPP3 transcript and protein levels decrease, leading to a reduction in pre-RNA processing and, consequently, a lower level of mtDNA translation [108]. The translational mtUPR axis complements the transcriptional canonical mtUPR axis in combating mitochondrial protein misfolding.

Thirdly, a sirtuin mtUPR axis has been reported. Sirtuins are lysine deacetylases that control several cellular processes. In mammals, there are seven sirtuins: SIRT1, 6, and 7, primarily localized in the nucleus; SIRT2, which predominantly localizes in the cytosol; and SIRT3, 4, and 5, primarily present in mitochondria [111,112,113]. The roles of all sirtuins in the mtUPR have been described [114]. However, SIRT1 and SIRT3 have been reported to have predominant functions. These sirtuins exert an antioxidant effect by deacetylating FOXO3A, which then translocates to the nucleus and promotes the transcription of antioxidant enzymes such as catalase and mitochondrial superoxide dismutase 2 (SOD2) [115,116]. As NAD^+^ levels decline with mitochondrial dysfunction and reduced NAD^+^/NADH ratios are implicated in mitochondrial disorders, NAD^+^-boosting agents (niacine, nicotinamide riboside, etc.) may induce SIRT activation, which promotes the antioxidant response [117]. Moreover, it has been proposed that different mitochondrial stressors, including the accumulation of misfolded and unfolded protein within the mitochondrial matrix, trigger the activation of SIRT3, resulting in a sirtuin mtUPR axis complementary to the transcriptional and translational canonical mtUPR axes [118].

Finally, another mtUPR axis has been reported; it is activated when mitochondrial stress is localized in the IMS. This axis is mediated by estrogen receptor α (ERα), which is phosphorylated by Akt or increased ROS. Once activated, ERα stimulates NRF1, a critical factor in mitochondrial biogenesis, and OMI, an IMS-specific protease that degrades aberrant proteins, leading to activation of the IMS–mtUPR axis [119].

In contrast to the other mtUPR axes, which inhibit mitochondrial complex synthesis and promote mitochondrial assembly, the activation of the sirtuin or IMS mtUPR axes also induces mitochondrial biogenesis via SIRT1 and NRF1, respectively, thus increasing mitochondrial complex synthesis. In this way, two mitochondrial quality control mechanisms, mitochondrial biogenesis and mtUPR, act simultaneously to reinforce the restoration of mitochondrial function [97].

A representative figure of these four axes of the mtUPR can be found in the review by Cilleros-Holgado et al. [97]. In Table 1, we summarize the markers of the different mtUPR axes as well as the location where mitochondrial stress occurs in each of them.

Finally, it is important to consider that, in contrast to the mtUPR, mitophagy removes damaged mitochondria, reducing the mitochondrial population and consequently leading to shortages in ATP and cell death. Therefore, the mtUPR appears to be a more effective mechanism for preserving mitochondrial function [124]. Nevertheless, it is worth noting that the prolonged activation of the mtUPR can have detrimental effects due to the propagation of damaged mitochondria. To address this issue, mitochondria have a damage threshold at which they cease mtUPR activation and initiate mitophagy. This necessitates the precise regulation of both compensatory mechanisms to ensure proper cellular function [125].

## 3. mtUPR as a Therapeutic Target

Numerous studies support the therapeutic potential of mtUPR modulation for the treatment of a wide range of diseases, including primary and secondary mitochondrial diseases [87,97,126,127,128,129,130].

### 3.1. Primary Mitochondrial Diseases

In the case of primary mitochondrial diseases, those caused by mutations in nDNA or mtDNA genes that code for mitochondrial proteins, the activation of mtUPR leads to increased ATP production, the assembly of OXPHOS-related subunits, improved mitochondrial antioxidant capacity, and overall recovery of mitochondrial function [131]. For example, Perry et al. demonstrated that mtUPR activation using tetracyclines, especially doxycycline, improved the survival and proliferation of MELAS cybrids and benefitted Rieske disease (mitochondrial complex III knockout) mouse fibroblasts and *ND1* mutant cybrids under low-glucose conditions. They also tested the effect of other mtUPR activators such as pentamidine, known to inhibit mitochondrial translation, and retapamulin, which inhibits prokaryotic translation, and found that cell survival increased in all cellular models of mitochondrial diseases under nutrient stress conditions. Furthermore, the cited authors tested the effect of tetracyclines in a mouse model of Leigh syndrome lacking the mitochondrial complex I protein NDUFS4, resulting in improvements in body weight loss, longevity, and the amelioration of neurological decline [132]. Subsequently, Suárez-Rivero et al. confirmed the positive effect of tetracyclines and the consequent activation of mtUPR in fibroblasts derived from patients with mutations in the *GFM1* gene [133]. Suárez-Rivero et al. also described that the activation of mtUPR via pterostilbene and mitochondrial cofactors improved mitochondrial function in different cellular models of primary mitochondrial diseases, enhancing the sirtuin mtUPR and transcriptional canonical mtUPR axes [134].

Nevertheless, it is important to consider that the use of antibiotics and, especially, tetracyclines, for the treatment of mitochondrial diseases is controversial [135].

On the other hand, it has been reported that mtUPR activation in urine-derived stem cells obtained from patients with the m.3243A>G mutation characteristic of MELAS syndrome induced mitochondrial dysfunction, while its inhibition through silencing ATF5 resulted in an improvement in cellular pathophysiology via increasing mitochondrial membrane potential and reducing ROS levels [136]. This difference in outcomes could be due to varying levels of heteroplasmy that occur when mitochondrial diseases are caused by mtDNA mutations. For this reason, further studies are needed to confirm the effect of mtUPR activation on primary mitochondrial diseases.

### 3.2. Cardiac and Metabolic Disorders

In cardiac disorders, the mtUPR plays a protective role [137]. Wang et al. observed that treatment with oligomycin or doxycycline in mice was beneficial with respect to cardiac ischemia–reperfusion injury, reducing infarct size. However, for this treatment to be effective, ATF5 and therefore mtUPR activation was required [138]. In addition, Smyrnias et al. proposed that mtUPR induction through six different methods, namely, treatment with paraquat, treatment with isoproterenol (a β-adrenoreceptor agonist), treatment with G-TPP (a chaperone inhibitor), mutation of the ornithine transcarbamylase protein, treatment with Olaparib (a PARP inhibitor), or treatment with nicotinamide, improved mitochondrial respiration in stressed cardiomyocytes in mice and attenuated contractile failure and hypertrophy [139]. Similarly, Xu et al. demonstrated that choline treatment in a rat model of heart disease activated the sirtuin mtUPR axis and inhibited both myocardial metabolic dysfunction and myocyte hypertrophy [140]. Likewise, Wang et al. showed that mtUPR induction via oligomycin had a protective role in septic cardiomyopathy induced by lipopolysaccharide, reducing cardiac dysfunction and mitochondrial damage [124]. Furthermore, Zhang et al. reported that the activation of mtUPR via treatment with tetrahydrocurcumin in mice subjected to transverse aortic constriction surgery reduced cardiac hypertrophy and surgery-induced oxidative stress [141]. Nevertheless, as Smyrnias et al. described, the activation of the mtUPR is not only beneficial when induced by external agents, as cells themselves activate this compensatory pathway to reduce damage. Myocardial cells derived from patients with aortic stenosis exhibited high levels of mtUPR markers such as ATF5, Hsp60, or LonP1. Those with even higher levels showed reduced rates of cardiomyocyte death, decreased levels of abnormal fibrosis, and fewer markers of cardiac damage in plasma [139].

On the other hand, metabolic diseases are related to alterations in OXPHOS gene expression and general mitochondrial dysfunction [142]. The most widely studied metabolic disease is diabetes, characterized by high levels of glucose in the blood and insulin resistance [143]. Elevated blood sugar levels over time inhibit the function of chaperones and proteases, leading to the accumulation of aberrant proteins and protein aggregates [144]. For this reason, the activation of the mtUPR could be beneficial in the treatment of diabetes. Wardelmann et al. observed that treatment with intranasal insulin activated mtUPR in insulin-deficient mice, improving mitochondrial function, inhibiting autophagy, and preventing weight gain under high-fat diet conditions [145]. In addition, Lee et al. demonstrated that LonP1 protease deficiency promoted liver gluconeogenesis and insulin resistance, while its overexpression mitigated liver insulin resistance induced by treatment with cholesterol and palmitate in liver SK-HEP-1 cells derived from humans [146]. Moreover, Wu et al. suggested that high-fat or high-glucose diets increased ClpP protease expression in pancreatic Min6 β-cells, leading to a decrease in cell apoptosis and ROS production, as well as the stabilization of insulin signaling [147]. Also, Kleinridders et al. reported that patients and mice with type 2 diabetes had lower Hsp60 expression levels in the brain, and this phenomenon was associated with insulin resistance [148].

mtUPR modulation also provides protective effects in the metabolic liver disease setting. These pathologies are associated with alterations in OXPHOS and elevated ROS production. Gariani et al. described that treatment with nicotinamide adenine dinucleotide in mouse models of fatty liver disease reverted non-alcoholic fatty liver disease by activating SIRT1 and SIRT3, ultimately increasing hepatic β-oxidation and OXPHOS activity [149]. In addition, the induction of the sirtuin mtUPR axes via nicotinamide precursor supplementation attenuated body weight gain and fat mass among mice fed a high-fat diet [150].

### 3.3. Cancer

Finally, mtUPR modulation has also been proposed as a therapeutic target for the treatment of different types of cancer. However, in this case, the therapies are not based on the activation of this stress response but on its inhibition, as it has been shown that mtUPR markers such as ATF5, Hsp60, Hsp70, ClpP, or LonP1 are upregulated in a wide variety of cancers. There are many studies on this subject, which can be consulted in the following references: [151,152,153,154,155,156,157,158,159,160,161,162,163,164,165,166,167,168,169,170,171,172,173,174,175,176,177,178,179,180,181,182,183,184].

In conclusion, a substantial body of literature supports the modulation of mtUPR as a therapeutic target in the treatment of various diseases, all of which have mitochondrial dysfunction as a primary underlying cause. We will emphasize the utilization of this approach in the context of ageing and age-related diseases, particularly neurodegenerative disorders. These conditions have a significant societal impact, with a growing number of affected individuals and a notable lack of curative treatments.

### 3.4. Ageing and Neurodegenerative Diseases

Neurodegenerative diseases encompass a diverse group of disorders characterized by the progressive and selective loss of anatomically or physiologically connected neuronal systems [185].

A major risk factor for neurodegenerative disorders is the process of ageing. It has been hypothesized that mitochondria play a central role in this context by producing ROS and accumulating mutations in mtDNA, both of which are believed to accelerate the physiological ageing process [14]. It is well established that during ageing, mtDNA mutations accumulate, contributing to the decline in mitochondrial function [186]. mtDNA is more vulnerable to damage due to the absence of histones and other chromatin proteins as well as its proximity to sites of ROS production [187]. Furthermore, ROS imbalance has a significant impact on cognitive loss associated with ageing [188]. The brain is particularly susceptible to oxidative damage due to its high oxygen consumption rate, high concentration of extremely peroxidizable unsaturated fatty acids, and relative lack of antioxidant enzymes compared to other organs [189]. Additionally, ageing is accompanied by a reduction in mitophagy, which leads to the accumulation of damaged mitochondria, increased oxidative stress, and the induction of apoptosis [190].

Given the pivotal role of mitochondrial dysfunction in ageing, it is not surprising that mitochondrial quality control mechanisms play a crucial role in this process. Increasing mitochondrial biogenesis by activating SIRT1 has been shown to delay ageing and extend lifespan in animal models [152,191]. In line with this, numerous studies suggest that the induction of the mtUPR can extend lifespan. For instance, the lifespan of a complex IV mtETC mutant *C. elegans* specimen exceeded that of control worms, and this extension was attributed to the activation of mtUPR, as evidenced by elevated markers of this stress response, such as Hsp6 and Hsp10 [95]. Moreover, mtUPR activation through mitochondrial translation inhibition, resveratrol, or PARP inhibitors promoted longevity among worms [192]. Moreover, supplementation with NAD^+^ precursors or the inhibition of NAD^+^-consuming enzymes also prolonged lifespan in *C. elegans* [193]. Importantly, *C. elegans* is not the sole model organism in which the activation of the mtUPR is beneficial for ageing. In mice, damage to mitochondrial ribosomes and the subsequent mtUPR induction also delayed ageing [194]. Furthermore, in *D. melanogaster*, mtUPR stimulation via the overexpression of the TRAP1 chaperone promoted both longevity and healthspan [195]. This beneficial effect was also observed in the fungal ageing model *P. anserina*, where the overexpression of the mitochondrial protease LonP1, a marker of mtUPR, extended lifespan [196].

In summary, there is sufficient evidence to suggest that the activation of the mtUPR can be helpful in delaying ageing and prolonging lifespan and that it may also play a beneficial role in the treatment of neurodegenerative diseases, as discussed below.

#### 3.4.1. Alzheimer’s Disease

Alzheimer’s Disease (AD) is the most prevalent form of neurodegeneration, affecting more than 47.5 million people worldwide. AD is characterized by two neuropathological hallmarks: (1) neurofibrillary tangles formed by neurofilaments and hyperphosphorylated tau protein and (2) extracellular senile plaques formed via the accumulation of amyloid β (Aβ) peptide. Together, these hallmarks result in an irreversible loss of neurons, especially in the cortex and hippocampus, leading to memory and cognition impairments. The majority of AD cases occur sporadically among individuals over 65 years of age [197].

**Figure 5:**
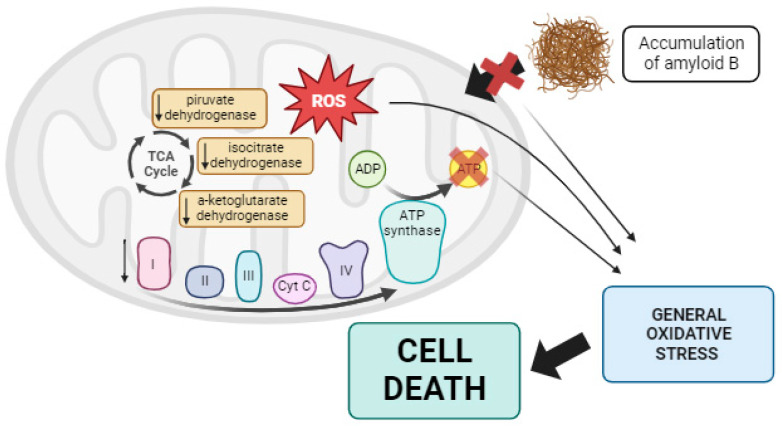
Summary of mitochondrial dysfunction in AD. In Alzheimer’s disease, lower activity of some TCA enzymes such as pyruvate dehydrogenase, isocitrate dehydrogenase, and α-ketoglutarate dehydrogenase has been observed, along with alterations in the mtETC. These changes collectively result in reduced ATP production and the overproduction of ROS. Additionally, this promotes the aberrant processing of APP, leading to the accumulation of Aβ, which disrupts the import of mitochondrial proteins into the mitochondria. All of these factors contribute to generalized oxidative stress in the cell, ultimately causing cell death via apoptosis. Arrow: activation or induction of the subsequent process.

Numerous studies support the role of mitochondrial dysfunction in the pathogenesis of AD. For instance, the treatment of pig neurons with hydrogen peroxide increased intracellular Aβ levels [198], and treatment with CCCP caused intracellular Aβ accumulation in cultured astrocytes [199]. It has been suggested that oxidative damage precedes Aβ deposition in animal models [200]. Additionally, post-mortem analysis of AD brains has revealed impaired activities of three essential mitochondrial enzymes: the pyruvate dehydrogenase complex, the α-ketoglutarate dehydrogenase complex, and isocitrate dehydrogenase [201,202,203]. Moreover, defects in complexes of the mtETC have been reported in AD cases [204]. These mtETC abnormalities reduced ATP production and increased ROS [205]. Moreover, mitochondrial dysfunction, along with the resulting ROS overproduction, may alter amyloid precursor protein (APP) processing, leading to the intracellular accumulation of Aβ, a characteristic feature of AD [198]. Aβ aggregation can block the import of nuclear-encoded mitochondrial proteins, causing oxidative damage to mitochondrial and cellular proteins, nucleic acids, and lipids. This sets off a vicious cycle, ultimately resulting in apoptosis and cell death [206,207]. Moreover, mitochondrial size and shape are altered in AD patients, with mitochondria exhibiting an elongated, interconnected appearance referred to as “mitochondria on a string” [208]. This is linked to disruptions in mitochondrial dynamics, particularly reduced mitochondrial fission and mitophagy, as noted by Zhang et al. [209] (see Figure 5).

Considering the pivotal role of mitochondria in AD, the mtUPR could be a potential therapeutic target for this disease. Indeed, Beck et al. demonstrated an increase in the expression levels of mtUPR-associated proteins in the frontal cortex of sporadic AD patients [210]. This finding has also been reported by other authors, such as Sorrentino et al., who observed an upregulation of mtUPR-associated genes in AD patients, mouse models, and *C. elegans* as a protective response during disease progression [211]. Moreover, in an AD *C. elegans* model, *ATFS-1* depletion via RNAi led to severe cognitive impairment, reduced mitochondrial respiration, and exacerbated Aβ aggregation, highlighting the protective role of the mtUPR in this disease [211]. Treatment with doxycycline or nicotinamide precursors to induce the mtUPR in the cited worm model and in mice improved overall health and lifespan by enhancing both animal motility and the clearance of Aβ peptides [211]. These authors also tested the positive effect of doxycycline on SHSY5Y cells with an APP mutation, observing a reduction in Aβ deposits and an improvement in AD pathophysiology [211]. Using the CL2006 *C. elegans* model of AD, which expresses the pathologic Aβ42, Regitz et al. demonstrated that treatment with resveratrol, a mtUPR activator, decreased Aβ aggregation and mitigated worm paralysis [212]. Similarly, Pérez et al. observed the induction of the mtUPR in *pitrylisin metallopeptidase 1* (*PITRM1*) mutant cortical neurons obtained from induced pluripotent stem cells, as shown by increased levels of ATF4, CHOP, Hsp60, ClpP, and LonP1. These mutant neurons exhibited increased levels of Aβ peptides without aggregation, possibly due to the protective role of the mtUPR. Inhibition of the mtUPR with ISRIB, an inhibitor of eif2α phosphorylation, resulted in the appearance of Aβ deposits and increased cell death [213]. Likewise, the treatment of SHSY5Y cells with Aβ caused the activation of Hsp60, ClpP, and OMI. In fact, the activation of OMI protease was also detected in various human brain regions postmortem [214]. In the brains of *APPsw/PS1De9* double transgenic mice, mtUPR induction was also demonstrated [215]. Furthermore, the activation of the mtUPR via nicotinamide riboside or olaparib restored mitochondrial membrane potential and morphology, increased mtDNA content, reduced Aβ peptide aggregation, and improved muscle integrity and fitness in *C. elegans* [216].

Considering all the studies published to date and the activation of the mtUPR in several Alzheimer’s disease models, it is evident that the induction of this mitochondrial stress response with external agents could be an effective strategy for the treatment of AD.

#### 3.4.2. Parkinson’s Disease

Parkinson’s Disease (PD) is the most prevalent movement condition and the second-most common neurodegenerative disease, affecting more than 2% of the population over the age of sixty worldwide [217]. This disorder is characterized by two hallmark features: (1) the presence of intraneuronal cytoplasmic aggregated α-synuclein protein inclusions known as Lewy bodies, and (2) a reduction in dopamine levels in the basal ganglia due to the death of dopaminergic neurons in the substantia nigra pars compacta, located in the midbrain [218]. Several studies have suggested that mitochondrial dysfunction plays a role in the pathophysiology of PD. Mutations in genes such as *Parkin* or *PINK1* are responsible for early-onset autosomal recessive PD, highlighting the importance of mitochondria in the disease’s etiology [219,220]. Mutations in the *DJ-1* gene, which controls the NRF2 transcription factor involved in mitochondrial biogenesis [221], and mutations in the OMI protease involved in the IMS-mtUPR [222] have also been linked to PD.

However, most PD cases are not genetic but sporadic, and in the sporadic form of the disease, mitochondria are still implicated. Exposure to compounds such as MPTP or rotenone, which are inhibitors of mitochondrial complex I, can induce a parkinsonian phenotype, lead to the degeneration of dopaminergic neurons, and result in the occurrence of α-synuclein inclusions [223,224,225]. Additionally, a reduction in complex I activity has been found in the brains of PD patients, establishing a link between complex I inhibition and sporadic PD [226]. Moreover, inherited mtDNA mutations can also cause parkinsonism [227], and PD patients have been found to have increased levels of somatic mtDNA deletions [228]. Furthermore, according to some findings, oxidative stress and the presence of ROS may be among the primary causes of PD, as increased levels of oxidized lipids, proteins, and DNA have been detected in the substantia nigra of PD patients. In addition to ROS, reactive nitrogen species (RNS) also play an important role in PD. Nitric oxide (NO) is generated by nitric oxide synthase (NOS) and is present in high concentrations in cells and in the extracellular space surrounding dopaminergic neurons, as demonstrated in postmortem brain tissue from PD patients [229]. NO can interfere with various enzymes and complexes I and IV, leading to increased ROS production. It can also cause lipid peroxidation and disrupt the proper functioning of proteins [230,231].

**Figure 6:**
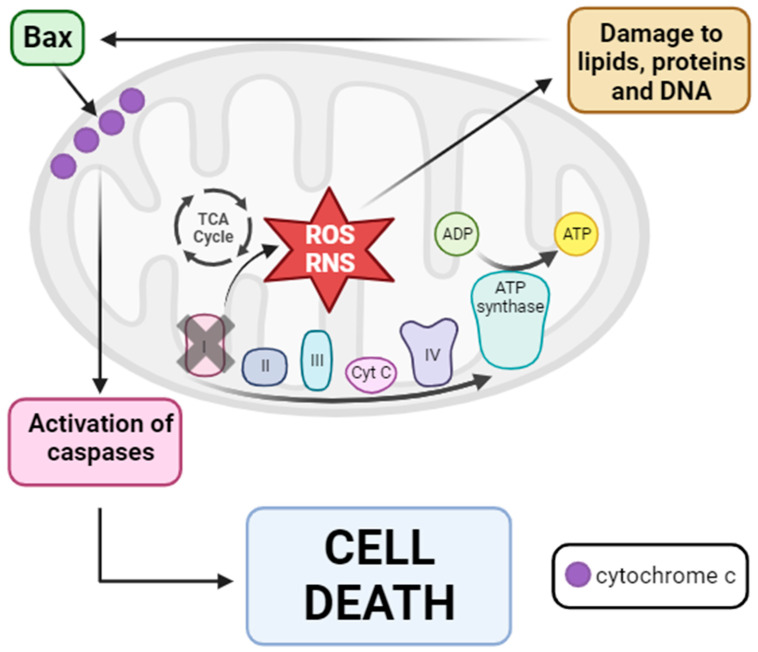
Mitochondrial dysfunction hypothesis of PD pathogenesis. Inhibition of mitochondrial complex I provokes the disruption of mtETC with consequent ROS and RNS production. Then, these reactive species damage several cellular components such as lipids, proteins, and DNA, causing the entry of Bax from the cytosol into the OMM. Once in the OMM, Bax provokes the release of cytochrome c into the cytosol, leading to the activation of the caspase signaling pathway and finally cell death via apoptosis. Arrow: activation or induction of the subsequent process.

Based on these findings, a hypothesis regarding mitochondrial dysfunction in PD pathogenesis has been proposed. The inhibition of complex I disrupts electron flux through mtETC, leading to increased production of ROS and RNS. These reactive species damage different cellular components, including lipids, proteins, and DNAactivating proapoptotic pathways with the entry of Bax into the mitochondria. Bax subsequently causes the release of cytochrome c into the cytosol, ultimately activating the caspase signaling pathway and resulting in cell death via apoptosis [232] (see Figure 6). 

Given this hypothesis, it is not surprising that the activation of mitochondrial quality control mechanisms may act as a therapeutic target for Parkinson’s disease. Regarding the mtUPR, Cooper et al. showed that in mutant *PINK1*, *Parkin*, and *DJ-1 C. elegans* PD models, mtUPR activation occurred, which protected worms from the death of dopaminergic neurons [233]. Dastidar et al. reported that overexpression of 4E-BP1, an inhibitor of eukaryotic initiation factor 4E and protein translation, protected animals against neurotoxicity induced by rotenone and paraquat. This protection was attributed to the activation of the mtUPR, as indicated by increased levels of CHOP, ATF4, Hsp60, ClpP, and MnSOD [234]. When CHOP was silenced, neuroprotection did not occur, and increased neuronal death was observed [234]. Finally, Liu et al. proposed that, in a *D. melanogaster* PD model, treatment with Ginseng, a Chinese herbal medicine, alleviated dopaminergic neuron death, improved locomotor function, and extended lifespan. These authors suggested that the beneficial effect of Ginseng was mediated via mtUPR enhancement, as they observed a significant induction of mtUPR-related genes such as Hsp60, Hsp70, and ClpP [235].

Nonetheless, it is important to note that overactivation of the mtUPR, such as through the elimination of MTS of ATFS-1 in a *C. elegans* PD model, provoked the death of dopaminergic neurons in a non-apoptotic manner, worsening the disease phenotype [236]. For this reason, while mtUPR induction may be effective in the treatment of Parkinson’s disease, the levels of activation should be carefully controlled, as overactivation might have detrimental effects. Further research is needed to fully understand the optimal level of mtUPR activation for therapeutic benefit.

#### 3.4.3. Huntington’s Disease

In Huntington’s Disease (HD), the role of mitochondrial dysfunction has also been proposed. HD is characterized by the progressive loss of GABAergic medium spiny neurons in the striatum, although degeneration is also observed in other brain regions such as the thalamus, the subthalamic nucleus, white matter, or the cerebellum [237]. This disorder affects 1 in 10,000 people worldwide [238]. Unlike Alzheimer’s and Parkinson’s diseases, where most cases are sporadic, the cause of HD is genetic [239]. This disease is caused by a CAG triplet expansion in the *huntingtin* gene, resulting in an expansion of polyglutamine in the N-terminal end of the protein. A number greater than 40 repeats is considered pathogenic, while the normal number is less than 36 [240]. 

It has been reported that mutant huntingtin (mtHtt) causes defects in mitochondria, leading to neurotoxicity, neuronal dysfunction, and finally cell death [241]. Indeed, it has been demonstrated that mtHtt interacts with several proteins. In the nucleus, mtHtt binds to p53, a tumor suppressor that, in response to stress, activates Bax and thus cell death via apoptosis [242]. The inhibition of p53 in *D. melanogaster* and *M. musculus* HD models prevented cellular dysfunction and neurodegeneration [243]. In addition, mtHtt interacts with CREB and CREB binding protein (CBP), inhibiting their function and therefore mitochondrial biogenesis [244]. In mitochondria, mtHtt activates the Drp1 protein, leading to mitochondrial fragmentation and general mitochondrial dysfunction [245]. Another protein with which mtHtt interacts is valosin-containing protein (VCP), an ATPase associated with OMM protein turnover and parkin-dependent mitophagy. As a result, mtHtt–VCP interaction promotes increased mitophagy and neuronal degeneration associated with mitochondrial dysfunction [246]. The involvement of mitochondrial dysfunction has been confirmed, as decreased mitochondrial complex I, II, III, and IV activity has been reported in the brains of HD patients [241,247], along with impaired mitochondrial respiration and ATP production [248]. Moreover, the inhibition of mitochondrial complex II by 3-nitropropionic acid or malonate leads to a pathological phenotype that resembles HD [249]. Impaired succinate dehydrogenase activity has also been observed in postmortem HD brains, along with reduced mitochondrial membrane potential in HD patients’ muscles and lymphoblasts [241]. This could be due to the interaction of mutant huntingtin with the OMM, which causes mitochondrial membrane potential reduction and potentiates mitochondrial permeability transition [250]. Finally, mtDNA deletions have been identified in HD patients during disease progression [251].

**Figure 7:**
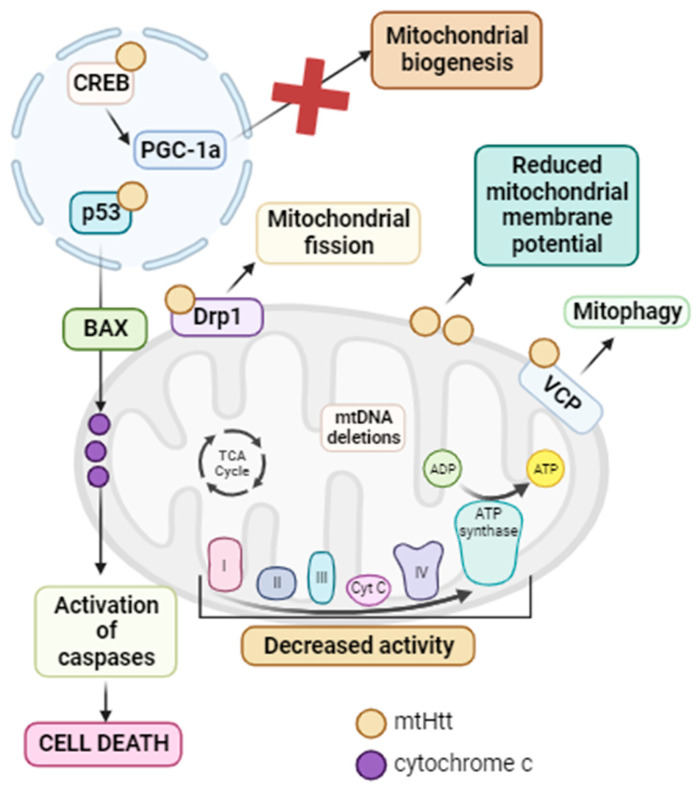
Summary of mitochondrial dysfunction in HD. mtHtt interacts with a large number of proteins. In the nucleus, it interacts with p53, promoting the activation of Bax with the subsequent release of cytochrome c from the mitochondria and the activation of caspases, ultimately leading to cell death via apoptosis. Additionally, in this same cellular compartment, mtHtt interacts with CREB, inhibiting it and thus suppressing PGC-1α and mitochondrial biogenesis. In the mitochondria, mtHtt interacts with Drp1, causing its activation and consequent mitochondrial fragmentation. Moreover, mtHtt binds to the OMM, leading to a decrease in mitochondrial membrane potential. It also interacts with VCP, activating mitophagy. All of these processes, along with the decreased activity of mtETC complexes and the accumulation of mtDNA deletions, contribute to the mitochondrial dysfunction characteristic of HD. Arrow: activation or induction of the subsequent process.

In summary, mtHtt interacts with a large number of proteins and mitochondrial structures such as the OMM. This interaction results in a reduction in mitochondrial biogenesis and membrane potential. It also leads to the opening of the mitochondrial permeability transition pore, calcium homeostasis failure, the induction of mitophagy, and mitochondrial fission. Additionally, there is a decrease in mitochondrial complex activity, elevated ROS production, and, ultimately, cell death, provoking the neurodegeneration characteristic of HD [252]. For this reason, the activation of mitochondrial quality control mechanisms and especially the mtUPR could be a potential treatment since mtHtt undergoes misfolding and proteolytic cleavage, resulting in N-terminal fragments that can form aggregates (see Figure 7). 

Accordingly, Fu et al. related that ABCB10, a component of the mtUPR pathway, had decreased transcript and protein expression levels in an HD mouse model and in dermal fibroblasts of two HD patients [253]. Overexpression of ABCB10 in striatal cells of an HD mouse model induced CHOP expression and reduced fragmented mitochondria, ROS production, and cell death via apoptosis [253]. The protein and mRNA levels of two markers of the mtUPR, Hsp60 and ClpP, were significantly reduced in fibroblasts derived from two HD patients, suggesting that mtHtt inhibited mtUPR activation in sufferers of HD [253]. Moreover, Hernández et al. observed that the ATF5 transcription factor, the master regulator of the mtUPR, was sequestered in mtHtt aggregates in HD mice, *C. elegans*, and patients, mainly in the cortex and striatum in the case of mice and patients [254]. Almeida et al. also observed decreased levels of mtUPR markers in PC12 cells expressing mtHtt [255]. On the other hand, Naia et al. demonstrated that SIRT3 expression levels and activity were elevated in several in vitro and in vivo models of HD [256]. However, this upregulation is not sufficient to counteract the toxic effects of mtHtt. Overexpression of SIRT3 in HD mouse and *D. melanogaster* models, as well as in HD-patient-derived fibroblasts, resulted in increased mitochondrial membrane potential, the elevation of mitochondrial complex activity, and decreased mitochondrial fragmentation, leading to the improvement of HD pathophysiology [256]. Furthermore, these authors also tested the effect of resveratrol in an HD mouse model, with the result being the activation of mitochondrial biogenesis through SIRT1 induction [257]. In the striatal cells derived from this HD mouse model, the use of nicotinamide induced the activation of sirtuin mtUPR, provoking an increase in mitochondrial antioxidant capacity with a consequent increase in mitochondrial membrane potential [257]. Likewise, treatment with both resveratrol and nicotinamide in HD-derived lymphoblasts restored mitochondrial respiration, with increased mitochondrial biogenesis and ATP production [257]. The positive effect of resveratrol and nicotinamide was also tested in the HD mouse model, where the authors observed that the restoration of mitochondrial function alleviated neurodegeneration signs and improved motor function [257]. Furthermore, Fu et al. showed that the activation of mitochondrial SIRT3 via treatment with ε-viniferin in a PC12 HD cell model reduced oxidative stress, promoted mitochondrial biogenesis, and improved mitochondrial dysfunction [258].

In conclusion, mitochondrial dysfunction is strongly associated with the pathogenesis of HD. mtHtt interacts with many proteins, among which is ABCB10, involved in the mtUPR, or CREB implicated in mitochondrial biogenesis, resulting in an impairment in mitochondrial quality control mechanisms. Therefore, the use of compounds that induce these pathways may be a potential therapeutic option for the treatment of HD.

#### 3.4.4. Amyotrophic Lateral Sclerosis

Amyotrophic Lateral Sclerosis (ALS) is a fatal neurodegenerative disorder characterized by the progressive loss of upper and lower motor neurons, mainly in the spinal cord, brainstem, and motor cortex. With an incidence of approximately 2.5/per 100,000 people worldwide, ALS is the third-most-common neurodegenerative disease after AD and PD [259]. While the majority of ALS cases are sporadic, 10% have a genetic cause. Since the discovery of the first gene associated with ALS, *superoxide dismutase 1* (*SOD1*) [260], more than 20 other genes have been reported to correlate with its pathogenesis, including *matrin 3* (*MATR3*), *coiled-coil-helix-coiled-coil-helix domain-containing 10* (*CHCHD10*), *fused in sarcoma/translocated in liposarcoma* (*FUS*), *chromosome 9 open reading frame 72* (*C9orf72*), *TAR DNA binding protein 43* (*TDP-43*), and *OPTN*, among others [261].

Several studies have proposed a strong association between mitochondrial function and ALS pathogenesis. In the case of familial ALS, mutations in the *SOD1* gene alter its antioxidant capacity, leading to ROS accumulation that inactivates axonal transport, induces axonal degeneration, and causes SOD1 to bind to Bcl-2, inhibiting Bcl-2’s anti-apoptotic activity. This promotes cytochrome C release and mitochondrially initiated caspase activation. In addition, altered calcium homeostasis in mitochondria due to *SOD1* mutations provokes the activation of apoptotic pathways and motor neuron death [262]. *C9orf72* mutations are also associated with mitochondrial dysfunction since C9orf72 is an IMM-associated protein. C9orf72 regulates complex I assembly by interacting with the IMM-domain-containing 1 (TIMMDC1) protein. When C9orf72 is mutated, the result is reduced mitochondrial complex I activity, provoking mitochondrial dysfunction and ALS motor symptoms [263]. On the other hand, mutations in *TDP-43* cause an alteration in mitochondrial dynamics, with an increased expression of FIS1 and Drp1 and decreased levels of Mfn1, leading to mitochondrial fragmentation, which affects mitochondrial function, including mtETC activity, ROS production, and calcium homeostasis, resulting in neuronal apoptosis [264]. In addition, mutations in *OPTN* cause a disruption in mitophagy since this protein is the primary receptor for PINK1/Parkin-mediated mitophagy. This causes an accumulation of damaged mitochondria and, once again, mitochondrial dysfunction, with a consequent cascade of signaling that eventually leads to neuronal death [265]. *MATR3* mutations have been found in four families with ALS. This protein has RNA- and DNA-binding domains that regulate gene expression, and it forms a complex with TDP43 and FUS, contributing to the mitochondrial dysfunction described above [266]. Similarly, *CHCHD10* mutations cause mitochondrial dynamics and cellular bioenergetics disturbances, as ALS patients with mutations in this gene present a fragmented mitochondrial network [267]. Moreover, the CHCHD10 protein interacts with TDP43 in the nucleus, but when it is mutated, the interaction does not occur, and TDP43 translocates to the cytosol, causing synaptic damage [268]. Finally, in the case of *FUS* mutations, the protein encoded by this gene forms aggregates in the cytosol of motor neurons. Additionally, it has been described that mutant FUS protein targets mRNAs encoding mitochondrial respiratory chain proteins in the nucleus and in the mtDNA, causing OXPHOS deficiency, ROS overproduction, and general mitochondrial dysfunction [269].

It is important to emphasize that in all these cases, there is an aggregation of the mutated protein inside the mitochondrion, which disrupts OXPHOS and leads to ROS accumulation, Ca^2+^ dyshomeostasis, mitochondrial dynamics alterations, mitophagy failure, and mitochondrial dysfunction. These alterations alter axonal transport, induce axonal degeneration, and ultimately lead to neuronal apoptosis [270]. 

Regarding sporadic cases, even though they do not follow a family history, mutations in some of the ALS-related genes have been found in most cases. In Table 2, we summarized the principal genes associated with ALS and their pathogenic mechanisms.

It is therefore important to consider the role of mitochondria, and most notably mitochondrial dysfunction and oxidative stress, in the pathogenic mechanisms of ALS.

Interestingly, the mtUPR is also associated with ALS. Wang et al. observed mtUPR activation in TDP-43 ALS cellular and animal models. Moreover, they described that TDP-43 interacted with LonP1, leading to its degradation. LonP1 overexpression protected cells from TDP-43-induced neurotoxicity, reducing mitochondrial damage and neurodegeneration. In contrast, inhibiting this protease decreased cell viability, suggesting a protective role of the mtUPR in TDP-43 pathogenesis [271].

In addition, Deng et al. demonstrated that mutant FUS accumulated within mitochondria, where FUS aggregates interacted with the ATP5B subunit of the ATP synthase complex. This interaction reduced mitochondrial ATP synthesis in HEK293 cell lines expressing mutant FUS and in a FUS-ALS *D. melanogaster* model. As a result, mitochondrial impairment occurred, and the mtUPR was activated, evidenced by the upregulation of mtUPR-associated genes such as ATF5, LonP1, Hsp60, and Hsp70 in both ALS cellular and animal models [272]. However, in contrast to the TDP-43 mutation case, LonP1 overexpression in the FUS-ALS *D. melanogaster* model exacerbated retinal degeneration, with increased neurodegeneration, while silencing mtUPR-related genes ameliorated mutant FUS-induced neurodegeneration [272].

On the other hand, Zhou et al. reported that mtUPR activation in an ALS mouse model with an *SOD1* gene mutation, achieved via treatment with nicotinamide precursors, decreased mitochondrial dysfunction and SOD1 aggregates and improved adult neurogenesis [273]. Furthermore, Straub et al. observed elevated mtUPR marker levels for both the transcriptional canonical and sirtuin mtUPR axes in fibroblasts derived from mutant *CHCHD10* patients when cultured under galactose stress conditions [274]. Finally, Riar et al. described that in a mouse model of ALS carrying the G93A-mutation in the SOD1 protein, IMS-mtUPR axis activation was induced when mutant SOD1 accumulated in the IMS. Interestingly, the genetic ablation of ERα induced the activation of the transcriptional canonical mtUPR axis, suggesting that in the absence of ERα, cells induced the CHOP/ATF/ATF5 axis as a compensatory mechanism [275]. It is worth noting that the activation of both axes of the mtUPR was found to be sex-specific, with significantly higher levels of induction observed in female mice compared to male mice (for which the levels were low) [275].

In conclusion, the role of the mtUPR as a therapeutic target in ALS remains complex. Its activation shows varied effects, with some cases indicating positive outcomes due to the aggregation of mutated proteins, while others demonstrate deleterious effects, as observed with LonP1 overexpression. A potential avenue worth exploring is the induction of the sirtuin axis, as suggested by the positive effects seen in the treatment with nicotinamide precursors. However, it is important to note that this observation is based on limited studies, and further research is essential to elucidate the full therapeutic potential of mtUPR pathways in ALS.

#### 3.4.5. Friedreich’s Ataxia (FA)

The implications of mitochondrial dysfunction and the mtUPR have also been described in Friedreich’s Ataxia (FA), a rare disease arising from a GAA repeat expansion in the *frataxin* (*FXN*) gene, which encodes the mitochondrial FXN protein involved in iron-sulfur cluster biosynthesis [276]. Consequently, decreased protein expression levels of FXN result in mitochondrial dysfunction, characterized by reduced OXPHOS, ROS overproduction, abnormal mitochondrial morphology, and Ca^2+^ homeostasis alterations [277]. It has been reported that transcriptional canonical mtUPR axis markers such as ATF4 and CHOP were upregulated in a mouse model of FA [278]. 

In summary, the interplay between mitochondrial dysfunction, mtUPR activation, and its potential benefit for FA remains intricate. Ageing and neurodegenerative diseases exhibit a strong association with mitochondrial dysfunction, leading to various pathological events culminating in cell apoptosis. In response, cells activate their mitochondrial quality control mechanisms, primarily the mtUPR, in an attempt to restore mitochondrial function. However, in the majority of cases, the induction of this stress response is not sufficient as the diseases continue to progress. Therefore, activation by external agents may hold therapeutic promise, allowing cells to regain their capacity to combat mitochondrial damage and resulting in improvements in symptoms and disease physiopathology. This therapeutic potential has been demonstrated in various animal and cellular models across different neurodegenerative diseases, as discussed throughout this review. 

## 4. Conclusions

Mitochondrial function is essential for cell survival and overall fitness. In response to mitochondrial stress, cells activate distinct mitochondrial quality control processes based on the intensity of the stress. When faced with severe stress, the initial response involves inducing mitochondrial fission. If this fails to restore mitochondrial function, cells initiate mitophagy for the complete degradation of the damaged mitochondria. In case of moderate stress, mitochondria stimulate mitochondrial fusion and mtUPR mechanisms for the recovery of mitochondrial function. Four different axes of the mtUPR have been described, namely, transcriptional and translational mtUPR, sirtuin mtUPR, and IMS-mtUPR, which act in parallel and complementarily, working together to enhance the restoration of mitochondrial health.

On the other hand, the loss of mitochondrial homeostasis and proteostasis results in mitochondrial dysfunction, a core factor implicated in a wide range of pathologies. These include primary mitochondrial diseases, cardiovascular disorders, metabolic diseases, cancer, and neurodegenerative diseases, as well as a common physiological process: ageing. The activation of the different mtUPR axes has shown a beneficial effect in numerous models of these diseases. Notably, in cancer, the situation is the contrary, since the inhibition of this stress response is considered a therapeutic target. 

Therefore, modulating the mtUPR has emerged as a potential therapeutic target for the treatment of complex diseases that compromise quality of life and currently lack curative treatments. 

## Figures and Tables

**Table 1:** Summary of the different mtUPR axes with the locations where mitochondrial stress takes place and markers of each of these pathways.

mtUPR Axis	Location of the Mitochondrial Stress	Markers	References
Transcriptional canonical mtUPR	Mitochondrial matrix	eif2α CHOP ATF5 ATF4 Hsp60 Hsp10 Hsp70 ClpP LonP1	[120] [86] [98] [99] [85] [85] [85] [102] [120]
Translational canonical mtUPR	Mitochondrial matrix	MRPP3	[108]
Sirtuin mtUPR	Mitochondrial matrix	SIRT1 SIRT3 FOXO3A MnSOD Catalase	[116] [121] [121] [115] [122]
IMS mtUPR	Intermembrane space	ERα NRF1 OMI	[119] [119] [123]

**Table 2:** Summary of the main genes related to ALS and their pathogenic mechanisms. It is important to note that mitochondrial dysfunction is present in all cases.

ALS-Related Gene	Pathogenic Mechanism	References
*SOD1*	Antioxidant defense deficiency ROS accumulation Mitochondrial dysfunction Axonal transport disturbance Axonal neurodegeneration Apoptosis induction	[262]
*C9orf72*	Decreased complex I assembly and activity Reduced OXPHOS ROS overproduction Mitochondrial dysfunction	[263]
*TDP-43*	Increased Fis1 and Drp1 levels Decreased Mfn1 levels Mitochondrial network fragmentation Mitochondrial membrane potential loss Reduced mitochondrial ATP synthesis Mitochondrial dysfunction	[264]
*OPTN*	Decreased mitophagy Accumulation of damaged mitochondria Mitochondrial dysfunction	[265]
*MATR3*	Interaction with TDP-43 and FUS Mitochondrial dysfunction	[266]
*CHCHD10*	Mitochondrial dynamics alteration Cellular bioenergetics failure TDP-43 aggregates Mitochondrial dysfunction	[267,268]
*FUS*	Aggregates formation Decreased mitochondrial complexes mRNA transcription Reduced OXPHOS Increased ROS Mitochondrial dysfunction	[269]

## Data Availability

Not applicable.

## Abbreviations

Aβ	amyloid β
AD	Alzheimer’s Disease
ALS	Amyotrophic Lateral Sclerosis
AMBRA1	autophagy and beclin 1 regulator 1
AMPK	AMP-activated kinase
APP	amyloid precursor protein
ATF4	Activating Transcription Factor 4
ATF5	Activating Transcription Factor 5
ATFS-1	activating transcription factor associated with stress-1
BNIP3	BCL2/adenovirus E1B 19 kDa protein-interacting protein 3
C9orf72	chromosome 9 open reading frame 72
CaMK	calcium-calcium/calmodulin-dependent protein kinase
CHCHD10	coiled-coil-helix-coiled-coil-helix domain containing 10
CHOP	C/EBP Homologous Protein
CLPP-1	caseinolytic protease P
CREB	cAMP response element-binding protein
DELE1	DAP3 Binding Cell Death Enhancer 1
Drp1	dynamin-related protein 1
DVE-1	homeodomain-containing protein transcription factor “defective proventiculus”
eif2α	eukaryotic translation initiation factor 2α
ER	endoplasmic reticulum
ERα	estrogen receptor α
FA	Friedreich’s Ataxia
FIS1	mitochondrial fission 1 protein
FUNDC1	Fun14 domain-containing protein 1
FUS	fused in sarcoma/translocated in liposarcoma
HAF-1	inner-membrane-spanning ATP-binding casette transporter
HD	Huntington’s Disease
HSF1	heat shock transcription factor 1
IMM	inner mitochondrial membrane
IMS	intermembrane space
ISR	Integrated Stress Response
LC3	microtubule-associated protein 1A/1B light chain 3
LonP1	Lon protease-like 1
MATR3	matrin 3
MDVs	mitochondrial-derived vesicles
MFF	mitochondrial fission factor
Mfn1	mitofusin 1
Mfn2	mitofusin 2
MID49	mitochondrial dynamics protein 49
MID51	mitochondrial dynamics protein 51
mPOS	mitochondrial precursor overaccumulation stress
mtDNA	mitochondrial DNA
mtETC	mitochondrial electron transport chain
MTS	mitochondrial targeting signal
mtUPR	mitochondrial unfolded protein response
nDNA	nuclear DNA
NLS	nuclear localization signal
NRF1	nuclear respiratory factor 1
NRF2	nuclear respiratory factor 2
OMM	outer mitochondrial membrane
Opa1	Optic Atrophy 1
OPTN	optineurin
OXPHOS	oxidative phosphorylation
PD	Parkinson’s Disease
PGC-1α	peroxisome proliferator-activated receptor γ co-activator 1α
PINK1	PTEN-induced kinase 1
PITRM1	pitrylisin metallopeptidase 1
PKA	protein kinase A
PLEKHM1	pleckstrin homology domain-containing family M member 1
ROS	reactive oxygen species
SIMH	stress-induced mitochondrial hyperfusion
SLP-2	stromatin-like protein 2
SOD1	superoxide dismutase 1
SOD2	superoxide dismutase 2
SSBP1	single-stranded DNA binding protein 1
TAX1BP1	Tax1-binding protein 1
TCA	tricarboxylic acid
TDP-43	TAR DNA binding protein 43
TFAM	mitochondrial transcription factor A
TIM	translocase of the inner membrane
TIMMDC1	IMM-domain containing 1
TOM	translocase of the outer membrane
UBL-5	ubiquitin-like protein 5
UPRam	Unfolded Protein Response activated by mistargeting of proteins
UPR^ER^	Unfolded Protein Response of the endoplasmic reticulum
UV-C	ultraviolet C
VDAC1	voltage/dependent anion channel 1
